# mRNA Therapeutics for Skin Rejuvenation: From Aging Atlases to Clinical Translation

**DOI:** 10.1111/exd.70367

**Published:** 2026-09-27

**Authors:** Fabiana Boncimino, Stefano Sol, Kristina Todorova, Anna Mandinova

**Affiliations:** ^1^ Cutaneous Biology Research Center Massachusetts General Hospital and Harvard Medical School Charlestown Massachusetts USA; ^2^ Broad Institute of Harvard and MIT Cambridge Massachusetts USA; ^3^ Harvard Stem Cell Institute Cambridge Massachusetts USA

## Abstract

Skin aging is a multifactorial process characterized by the progressive decline of tissue structure, function and regenerative capacity. Although numerous interventions have been developed to improve the clinical manifestations of aging, most primarily alleviate downstream consequences rather than directly targeting the molecular mechanisms underlying tissue deterioration. Recent advances in single‐cell and spatial transcriptomic technologies have fundamentally transformed our understanding of skin aging by identifying cell type‐specific transcriptional programmes and uncovering novel regulators of epidermal homeostasis, dermal remodelling and intercellular communication. These high‐resolution atlases have generated an expanding repertoire of candidate rejuvenation targets, many of which consist of intracellular regulatory proteins that have traditionally been considered difficult to manipulate using conventional pharmacological approaches. In parallel, rapid progress in RNA engineering has established nucleic acid therapeutics as a versatile approach for precise molecular intervention. Among these, messenger RNA (mRNA) therapeutics are particularly well suited to the molecular landscape emerging from skin aging atlases because they enable transient restoration of proteins that are lost during aging. Advances in lipid nanoparticles, extracellular vesicles and microneedle‐based delivery systems are rapidly improving the feasibility of local cutaneous administration. In this review, we discuss the emerging rejuvenation targets identified by single‐cell and spatial transcriptomic atlases and examine recent advances in RNA‐based therapeutics and mRNA delivery strategies that are paving the way for precision skin rejuvenation.

**Abbreviations**
AAV: adeno‐associated virus
ASO: antisense oligonucleotide
CMC: chemistry, manufacturing and controls
dsRNA: double‐stranded RNA
EV: extracellular vesicle
LNP: lipid nanoparticle
miRNA: microRNA
MN: microneedle
mRNA: messenger RNA
PEG: polyethylene glycol
PNA: peptide nucleic acid
PRP: platelet‐rich plasma
RISC: RNA‐induced silencing complex
RNAi: RNA interference
SASP: senescence‐associated secretory phenotype
siRNA: small interfering RNA
UTR: untranslated region
UV: ultraviolet

## 1 Introduction

Skin aging is a complex process driven by the interaction between intrinsic mechanisms and lifelong environmental exposure [1, 2, 3]. Beyond visible manifestations such as wrinkles, laxity and pigmentation changes, it is associated with impaired barrier function, delayed wound healing, chronic low‐grade inflammation and increased susceptibility to age‐related skin disorders [3, 4, 5]. Developing effective rejuvenation strategies has therefore become a major goal in dermatological research and regenerative medicine [3, 5, 6].

Skin rejuvenation encompasses multiple dimensions, including restoration of tissue architecture, improvement of cellular and regenerative functions and reversal of age‐associated molecular alterations. Over the past decades, numerous approaches, including topical retinoids, energy‐based devices, growth factor therapies, senolytics and partial cellular reprogramming, have been explored to delay or reverse cutaneous aging [5, 6, 7, 8]. While several act through defined molecular pathways and improve clinical features of aged skin, most were developed before the cellular heterogeneity and cell type‐specific molecular alterations of skin aging were fully understood, limiting their ability to selectively target the biological processes driving tissue dysfunction.

Recent advances in single‐cell and spatial transcriptomics have fundamentally changed our understanding of skin aging by revealing previously unrecognized cellular heterogeneity, identifying cell type‐specific transcriptional programmes and uncovering novel molecular regulators of tissue homeostasis [9, 10, 11, 12]. In parallel, the rapid development of modified mRNA technologies and increasingly sophisticated delivery systems has created new opportunities to directly manipulate these rejuvenation‐associated pathways in a safe and transient manner [13, 14, 15].

In this review, we discuss how single‐cell aging atlases are reshaping the identification of therapeutic targets for skin rejuvenation and how mRNA‐based approaches are emerging as a versatile platform to translate these discoveries into next‐generation regenerative therapies. We also summarize current advances in cutaneous mRNA delivery, highlight the challenges associated with clinical translation and discuss future directions for precision skin rejuvenation.

## 2 Skin Aging

Skin aging is a gradual, multifactorial process characterized by progressive decline of tissue structure, function and regenerative capacity [1, 2], driven by environmental stress superimposed on intrinsic age‐dependent changes [2, 16]. Its visible manifestations, including wrinkles, laxity, dryness, pigmentary alterations and loss of elasticity, represent only the final outcome of a broader remodelling process [1]. Skin aging is therefore best understood as the cumulative result of interconnected alterations rather than a single dominant mechanism.

### 2.1 Intrinsic and Extrinsic Determinants of Skin Aging

Skin aging has traditionally been classified into two complementary processes: intrinsic (chronological) aging and extrinsic aging [2, 16].

Intrinsic aging reflects the genetically programmed decline associated with advancing age. Chronologically aged skin accumulates DNA damage, telomere shortening, epigenetic alterations, mitochondrial dysfunction, impaired proteostasis and stem cell exhaustion [1, 17], reducing the proliferative and regenerative capacity of epidermal and dermal cells while increasing cellular senescence [18, 19]. At the tissue level, intrinsic aging typically manifests as fine wrinkles, epidermal thinning, dermal atrophy, reduced elasticity and impaired barrier recovery [20, 21].

In contrast, extrinsic aging results from cumulative exposure to environmental and lifestyle factors [16]. Chronic ultraviolet (UV) radiation is the predominant driver of premature skin aging, accounting for most visible age‐associated changes in sun‐exposed skin [22, 23]. UV irradiation promotes DNA damage, oxidative stress, inflammation and matrix degradation while suppressing collagen synthesis and accelerating senescence [24, 25, 26]. Additional factors, including air pollution, smoking, poor nutrition, sleep disruption and environmental toxins, further amplify these processes [27, 28].

Rather than acting independently, intrinsic and extrinsic aging reinforce one another. Environmental insults accelerate molecular programmes normally associated with chronological aging, whereas age‐dependent deterioration of repair mechanisms progressively reduces the ability of the skin to manage external stress [2, 16, 29]. Consequently, the aged phenotype reflects the cumulative interaction between endogenous biological aging and lifelong environmental exposure.

### 2.2 Structural and Functional Alterations Across Skin Compartments

The biological consequences of aging affect all compartments of the skin, influencing not only individual cell populations but also tissue architecture and organ function (Figure 1).

**FIGURE 1:**
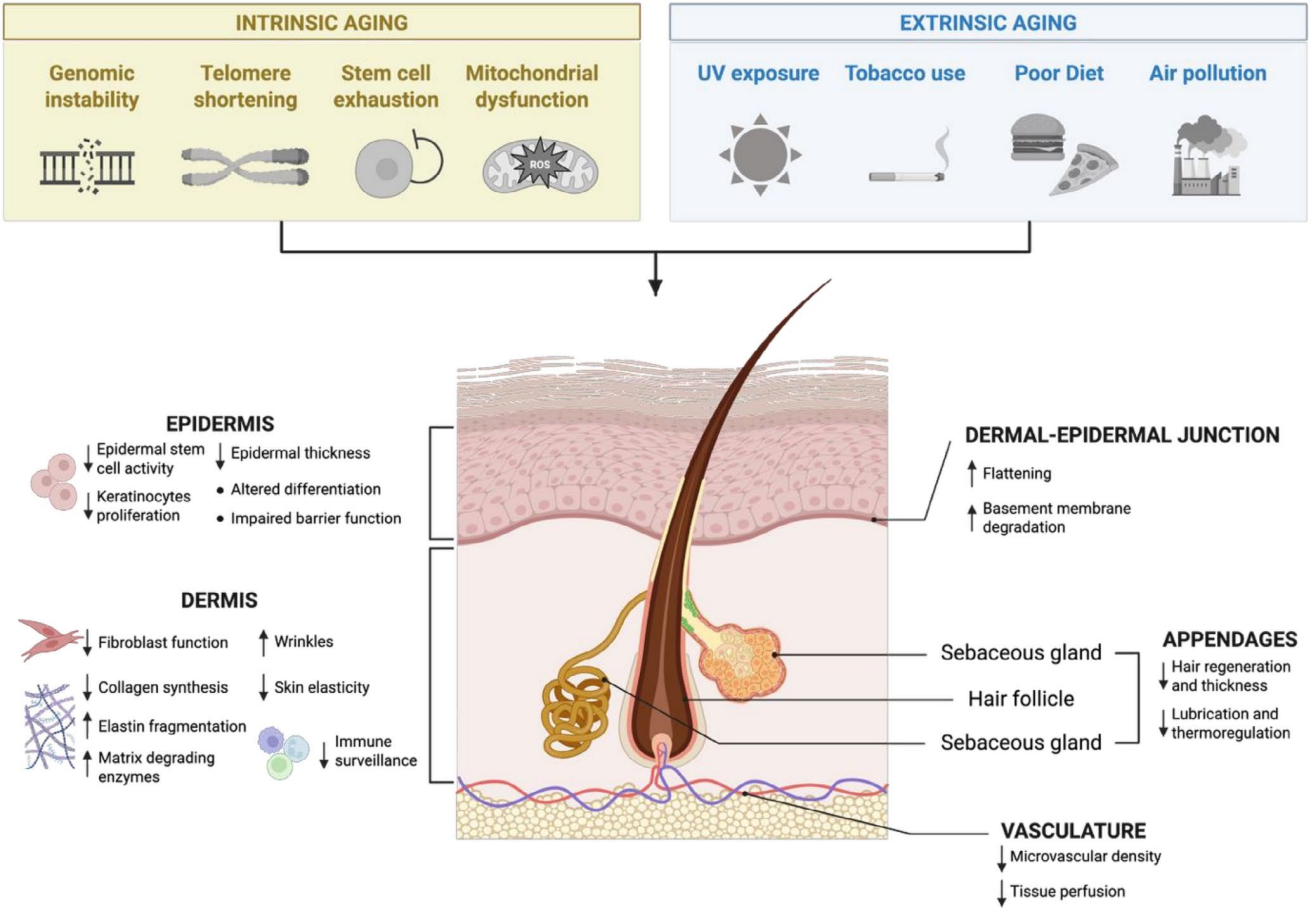
Hallmarks and tissue alterations associated with skin aging. Schematic overview of the major intrinsic and extrinsic drivers of skin aging and their effects on skin architecture. Intrinsic aging is characterized by genomic instability, telomere shortening, stem cell exhaustion and mitochondrial dysfunction, whereas extrinsic aging is primarily driven by chronic ultraviolet (UV) exposure together with environmental and lifestyle factors, including tobacco use, poor diet and air pollution. These processes progressively disrupt epidermal, dermal and appendage homeostasis, resulting in impaired epidermal renewal, reduced dermal extracellular matrix production, chronic low‐grade inflammation, flattening of the dermal‐epidermal junction and functional decline of skin appendages and the cutaneous vasculature. Image created using BioRender (BioRender.com).

Within the epidermis, aging brings a gradual decline in keratinocyte proliferation, driven by reduced epidermal stem cell activity and altered differentiation dynamics, resulting in progressive thinning [30, 31]. Flattening of the dermal‐epidermal junction reduces tissue mechanical stability and limits metabolic exchange with the underlying dermis [32], while age‐dependent impairment of lipid synthesis and barrier maintenance increases trans‐epidermal water loss, contributing to dryness, fragility, delayed healing and enhanced susceptibility to environmental insults [31, 33].

The dermis undergoes equally profound remodelling. Dermal fibroblasts lose their capacity to synthesize extracellular matrix proteins, resulting in reduced collagen production and fragmentation and disorganization of the extracellular matrix [34], while persistent activation of matrix‐degrading enzymes and impaired matrix renewal shift homeostasis toward net matrix loss [35, 36, 37]. These alterations ultimately manifest clinically as wrinkles, laxity, reduced elasticity and decreased mechanical strength [38].

Cutaneous appendages are similarly affected. Hair follicle stem cell function declines, contributing to hair thinning and delayed follicular regeneration [39, 40], while sebaceous and sweat gland activity decreases, impairing lubrication and thermoregulation [33, 41, 42]. Aging also reduces dermal microvascular density and perfusion [43, 44] and reshapes the cutaneous immune compartment, diminishing surveillance while promoting chronic low‐grade inflammation [45, 46, 47, 48, 49], together compromising the skin's ability to maintain homeostasis and respond to injury.

### 2.3 Skin Aging as a Multicellular Process

Although early studies primarily focused on isolated mechanisms such as oxidative stress, collagen degradation or fibroblast senescence, it is now evident that skin aging is fundamentally a multicellular process involving coordinated dysfunction across every cellular component of the tissue.

The epidermis, dermis, immune compartment and skin appendages continuously communicate through cell–cell interactions, extracellular matrix remodelling, soluble molecules and metabolic signals [50, 51, 52]. During aging, dysfunction within one compartment influences neighbouring populations, creating self‐reinforcing networks that promote tissue deterioration. Senescent cells secrete a senescence‐associated secretory phenotype (SASP) that alters surrounding epithelial, mesenchymal, endothelial and immune cells [53, 54]. Likewise, age‐dependent matrix remodelling alters the tissue microenvironment's biochemical and mechanical properties [34, 36, 37].

This integrated view has substantially changed our understanding of cutaneous aging. Rather than representing the simple accumulation of independent cellular defects, skin aging is increasingly recognized as the progressive disruption of tissue‐wide homeostasis, where alterations in individual cell populations become amplified through defective intercellular communication and microenvironmental remodelling.

Recognizing skin aging as a multicellular process has important therapeutic implications. Interventions aimed at a single pathway or a single cell type may improve selected aspects of tissue aging but are unlikely to fully restore tissue function if the broader cellular network remains dysfunctional. Consequently, identifying the specific cell populations and molecular circuits that orchestrate tissue aging has become a major objective of contemporary skin aging research.

## 3 Single‐Cell and Spatial Atlases Redefine Our Understanding of Skin Aging

Traditional cellular and histological approaches revealed many fundamental hallmarks of cutaneous aging, including epidermal thinning, matrix degradation, stem cell dysfunction, chronic inflammation and senescence, but provided only a limited view of the underlying cellular architecture. Single‐cell RNA sequencing and spatial transcriptomics transformed this landscape, enabling characterization of thousands of cells while preserving transcriptional identity and spatial organization [55, 56, 57], revealing specialized cell populations with distinct functional states and aging‐associated programmes [10, 11].

### 3.1 Single‐Cell Transcriptomic Atlases Reveal Cell Type‐Specific Aging Programs

An important consideration when comparing human skin aging atlases is how ‘aged’ skin is defined and how chronological aging is distinguished from cumulative environmental exposure. Current studies differ substantially in age ranges, anatomical sampling sites and approaches used to address intrinsic versus extrinsic aging (Table 1). These differences in study design should therefore be considered when interpreting and comparing age‐associated molecular signatures across studies.

**TABLE 1:** Human single‐cell and spatial transcriptomic studies of skin aging.

Study	Platform	Cohort size	Age	Sex	Ethnicity	Fitzpatrick skin type	Body site	Intrinsic vs. extrinsic aging	Key findings
Zou et al., 2021 [10]	10× Genomics scRNA‐seq (3′ v2)	9 donors (3 young +3 middle‐aged +3 old)	Young: 18–28; Middle‐aged: 35–48; Old: 70–76	Female only	Not reported	Not reported	Upper eyelid	Combined intrinsic and extrinsic; not experimentally separated	Aging induced broad transcriptional remodelling with increased inflammatory programmes and reduced DNA‐repair programmes; KLF6 in basal keratinocytes and HES1 in fibroblasts declined with age.
Solé‐Boldo et al., 2020 [11]	10× Genomics scRNA‐seq (v2)	5 donors (2 young +3 old)	Young: 25–27; Old: 53–70	Male only	Caucasian	Not reported	Sun‐protected inguinoiliac region	Intrinsic aging specifically assessed; photoaging minimized by sampling sun‐protected skin	Aging caused loss of fibroblast subtype identity, reduced collagen expression, increased inflammatory signatures and reduced predicted fibroblast interactions with other skin cell types.
Nakamizo et al., 2025 [58]	10× Genomics Chromium Next GEM scRNA‐seq (3′ v3.1)	6 donors (3 young +3 old)	Young: 20–30; Old: 60–80	Not reported	Not reported	Not reported	Upper eyelid	Combined intrinsic and extrinsic; not experimentally separated	Aged skin showed increased inflammatory/stress‐associated genes and reduced intercellular communication.
Ahlers et al., 2021 [59]	10× Genomics scRNA‐seq	7 donors (3 young +4 old)	Young: 22–29; Old: 67–80	Female only	Caucasian	Not reported	Outer forearm	Intrinsic aging primarily assessed; extrinsic not independently assessed	Aging was associated with loss of dermal sheath cells and reduced fibroblast stemness; dermal sheath‐derived Activin A promoted keratinocyte proliferation, epidermal thickness and procollagen production.
Melyukhina et al., 2026 [60]	scRNA‐seq	10 donors (5 young +5 aged)	Young: 31–36; Old: 72–77	2 M/8 F (young: 2 M/3 F; old: 5 F)	Not reported	Not reported	Young: abdomen (*n* = 4) + groin (*n* = 1); aged: abdomen (*n* = 3) + face (*n* = 2)	Intrinsic aging was the intended comparison, but anatomical site and sex differed between age groups; extrinsic effects were not independently controlled	Aged skin showed increased inflammatory programmes and reduced oxidative‐stress detoxification/mitochondrial function.
Li et al., 2026 [61]	Stereo‐seq spatial transcriptomics	4 donors (2 young +2 old)	Young: 18–24; Old: 51–71	Female only	Not reported	Not reported	Upper eyelid	Combined intrinsic and extrinsic; not experimentally separated	Aging was associated with dermal atrophy and chronic inflammation, reduced dermal–epidermal communication, impaired fatty‐acid metabolism and reduced epidermal c‐FOS/AP‐1 activity.
Yu et al., 2025 [62]	Integrative re‐analysis of published scRNA‐seq datasets (Solé‐Boldo et al.; Tabib et al.; Ganier et al.) and 10× Genomics Visium spatial transcriptomics (Ganier et al.)	scRNA‐seq: 26 samples; spatial: 22 samples	scRNA‐seq: 23–90; spatial: 33–77; no unified young‐vs‐old stratification	scRNA‐seq: 21 M/5 F; spatial: 19 M/3 F	Not reported	Spatial cohort: I/II for all samples	scRNA‐seq: dorsal mid‐forearm (*n* = 6), inguinoiliac (*n* = 5), face (*n* = 15); spatial: body (*n* = 9) + face (*n* = 13)	Both assessed, although not independently disentangled; chronological age and anatomical site/sun exposure are confounded and individual UV exposure was unavailable	Senescence‐associated signals were spatially non‐uniform; CDKN1A/p21‐high regions showed significant clustering in a subset of sun‐exposed samples, supporting localized senescence‐enriched niches.
Li et al., 2026 [63]	10× Genomics 3′ scRNA‐seq	8 donors (3 young +3 middle‐aged +2 old); 16 paired biopsies	Young: 23–28; Middle‐aged: 46–55; Old: 76–85	Female only	Not reported	Not reported	Paired arm and back skin from the same donors	Both assessed within donors: back used to emphasize intrinsic aging and arm to capture combined intrinsic and extrinsic	Aging was associated with reduced basal stem‐cell renewal; ATF3 emerged as a downregulated regulator.

Abbreviations: F, female; M, male; scRNA‐seq, single‐cell RNA sequencing.

A major breakthrough came with the first comprehensive single‐cell atlas of human skin aging, generated by Zou et al. from eyelid skin of young, middle‐aged and elderly donors [10], showing that chronological aging largely preserves overall cellular composition. Aging instead involved widespread transcriptional remodelling across nearly all cell types, with basal keratinocytes losing programmes governing development, proliferation, DNA repair and regeneration while acquiring inflammatory signatures. A prominent change was progressive downregulation of KLF6 across young, middle‐aged and older donors, whose inhibition in keratinocytes was sufficient to induce senescence, identifying it as a key regulator of epidermal homeostasis.

These findings were extended by a dedicated single‐cell analysis of the human epidermis, confirming that keratinocyte, melanocyte, Langerhans cell and lymphocyte abundance remain largely unchanged during aging despite extensive molecular remodelling [58]. Aged keratinocytes showed increased inflammatory mediators (CD55, RORA, MIF, SERPINB3), enrichment of oxidative phosphorylation and apoptotic signalling and computationally inferred reductions in intercellular communication, particularly from proliferative keratinocytes via BMP, FGF, kallikrein, melanocortin and pleiotrophin pathways, suggesting impaired cell–cell communication as a further mechanism of epidermal dysfunction.

Single‐cell transcriptomics identified dermal fibroblasts as among the populations with the most extensive transcriptional remodelling during aging [10, 11]. Solé‐Boldo et al. showed that aging preferentially disrupts the identity of each fibroblast subtype [11]. Papillary fibroblasts acquired reticular‐like features while reticular fibroblasts lost specialization—termed loss of fibroblast priming—indicating that aging blurs lineage‐specific programmes rather than reducing fibroblast number. Zou et al. found increased inflammatory and matrix‐degradation pathways in aged fibroblasts and identified *HES1* as one of the most consistently downregulated genes, contributing directly to dermal aging [10].

Subsequent studies further refined our understanding of fibroblast heterogeneity. Ahlers et al. identified dermal sheath cells as a previously underappreciated mesenchymal stem cell population that declines substantially during aging, secreting Activin A, which stimulates keratinocyte proliferation, increases epidermal thickness and enhances fibroblast collagen synthesis [59]. Their age‐dependent depletion suggested that loss of regenerative mesenchymal progenitors contributes to both dermal degeneration and impaired epidermal maintenance through paracrine signalling.

Additional single‐cell studies have broadened these observations. Liao and colleagues confirmed that aging induces a generalized decline in cellular activity across skin compartments together with predicted alterations in intercellular communication [64]. Computational ligand‐receptor analyses suggested that signalling between fibroblasts, keratinocytes, immune cells and vascular cells weakens with age, particularly across the dermal‐epidermal interface, reinforcing the concept that impaired cellular crosstalk is a fundamental feature of skin aging rather than a secondary consequence of extracellular matrix deterioration.

Importantly, single‐cell atlases have also demonstrated that aging extends far beyond keratinocytes and fibroblasts. Melanocytes acquire inflammatory and stress‐associated transcriptional programmes, potentially contributing to age‐related pigmentary alterations [10]. Endothelial cells exhibit increased expression of genes associated with vascular dysfunction and inflammation [10], whereas immune populations display features consistent with chronic low‐grade inflammatory activation and immune senescence [10, 60].

Collectively, these findings support the view that skin aging represents a coordinated multicellular process involving virtually every resident cutaneous cell type rather than isolated defects within individual compartments. Rather than being driven by widespread loss of specific cell populations, skin aging is associated with a remodelling of transcriptional programmes that affects all skin cell populations in distinct yet interconnected ways.

### 3.2 Spatial Transcriptomics Reveals Aging‐Associated Niches and Intercellular Communication

While single‐cell transcriptomics has characterized the transcriptional states of individual skin cell populations during aging, tissue dissociation inevitably disrupts the spatial relationships governing cell behaviour in vivo and may introduce cell‐type‐specific recovery biases and stress‐related transcriptional changes. Understanding how cells are arranged within aging tissue is equally important for deciphering mechanisms of skin degeneration. Spatial transcriptomic technologies therefore provide a complementary layer of information, enabling gene expression profiling while preserving tissue architecture, although current approaches remain constrained by platform‐dependent spatial resolution and, particularly for spot‐based approaches, mixed‐cell signals.

Using spatially enhanced‐resolution omics‐sequencing (Stereo‐seq) on human facial skin from young and elderly donors, Li et al. identified eight major spatial domains and gene modules linked to epidermal differentiation, dermal organization, immune regulation and lipid metabolism [61]. Aging involved profound remodelling of spatial gene expression, including reduced lipid‐metabolism genes *FASN* and *FDPS* with decreased c‐FOS/AP‐1 activity; inhibiting *FASN* and *FDPS* increased senescence. Independently, Yu et al. mapped senescent cell populations spatially, showing that senescence accumulates within discrete niches rather than uniformly [62].

Beyond identifying spatially restricted molecular programmes, spatial transcriptomics has improved our understanding of age‐dependent intercellular communication. Several studies have identified age‐associated changes in computationally inferred signalling between the epidermis and underlying dermis, with reduced coordination among fibroblasts, endothelial cells and immune populations [61, 64]. Rather than simply reflecting extracellular matrix deterioration, these observations suggest that the impairment of multicellular communication networks that normally maintain epidermal renewal, dermal matrix production and vascular function may potentially contribute to age‐associated loss of tissue homeostasis. Spatial transcriptomics thus extends single‐cell sequencing by revealing where age‐associated programmes arise.

## 4 Rejuvenation Targets Emerging From Aging Atlases

The emergence of single‐cell and spatial transcriptomic technologies, combining cellular identity with anatomical context, has facilitated the identification of rejuvenation targets selected not only by differential expression but also by their regulatory position within cell type‐specific transcriptional networks. These targets comprise diverse molecular classes, including transcription factors, metabolic enzymes and intercellular signalling molecules that can be experimentally deployed to improve distinct age‐associated molecular, functional and structural alterations (Figure 2).

**FIGURE 2:**
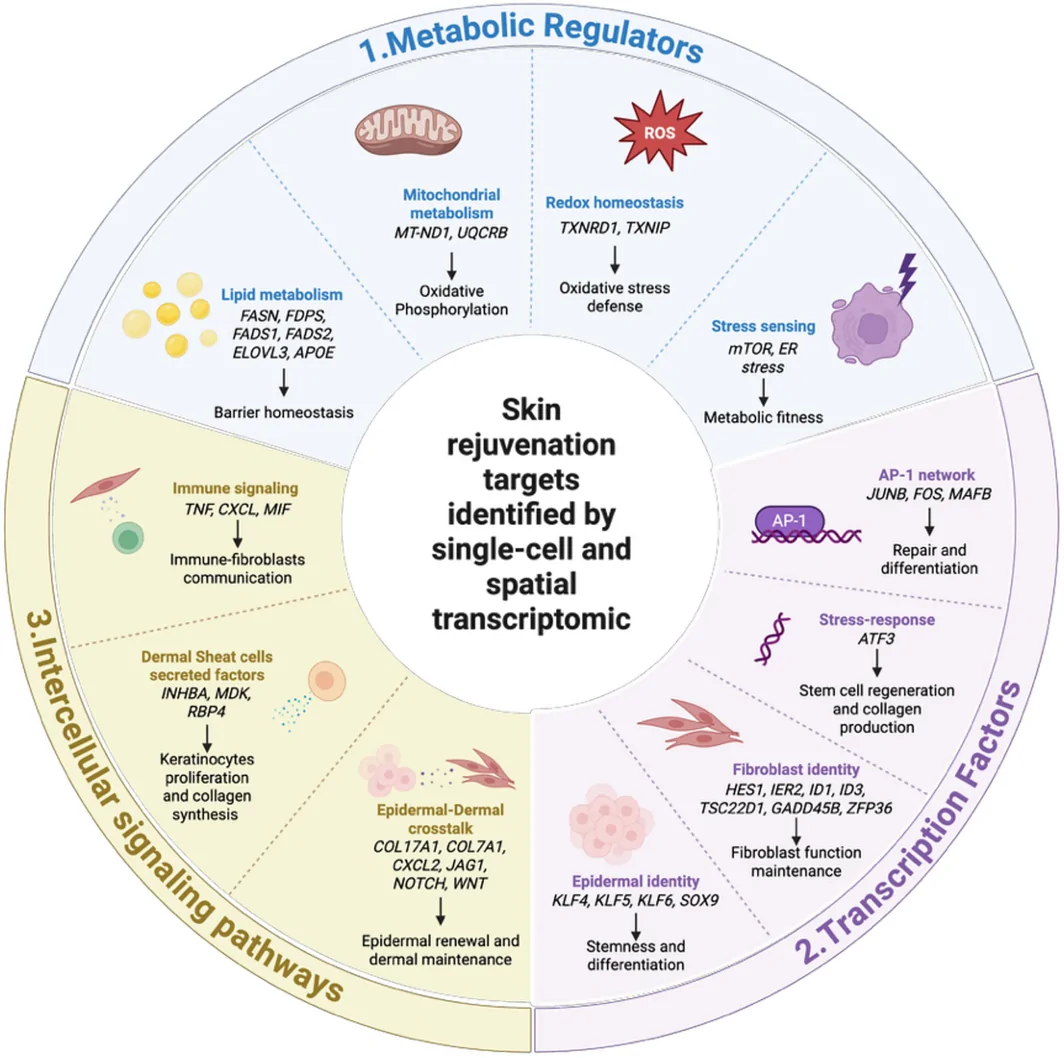
Candidate skin rejuvenation targets identified by single‐cell and spatial transcriptomic studies. Overview of the principal molecular pathways emerging from recent single‐cell and spatial transcriptomic studies as potential targets for skin rejuvenation. Candidate regulators were grouped into three major functional categories: Metabolic regulators, transcription factors and intercellular signalling pathways. Representative genes and signalling networks implicated in mitochondrial function, oxidative stress, epidermal and fibroblast identity, stress responses, immune–stromal communication, dermal sheath signalling and epidermal‐dermal crosstalk are shown together with their proposed biological functions in maintaining skin homeostasis and promoting tissue regeneration. Image created using BioRender (BioRender.com).

Single‐cell and spatial transcriptomic studies have identified numerous molecular alterations associated with skin aging. However, not all age‐associated genes represent suitable therapeutic targets. Many transcriptional changes likely reflect secondary consequences of tissue deterioration rather than causal regulators of aging. Prioritizing rejuvenation targets therefore requires integrating transcriptomic data with functional validation and biological relevance.

An ideal target should show reproducible age‐associated changes across independent studies and directly contribute to tissue homeostasis. Importantly, therapeutic modulation should restore physiological cellular function without disrupting normal differentiation or increasing oncogenic risk.

The candidate targets identified to date comprise several functional classes, including metabolic regulators, secreted signalling molecules and transcription factors. Secreted factors may simultaneously influence multiple cell populations through paracrine signalling, whereas metabolic regulators may improve fundamental aspects of cellular fitness. Transcription factors occupy a unique position because they coordinate gene networks controlling proliferation, differentiation and tissue regeneration. Their restoration therefore has the potential to normalize multiple age‐associated pathways simultaneously.

The multicellular nature of skin aging further suggests that effective rejuvenation strategies will require restoration of intercellular communication rather than correction of isolated molecular defects. Integrating single‐cell and spatial transcriptomics with functional studies will therefore be essential for distinguishing biomarkers of aging from molecular drivers that can be therapeutically targeted.

Candidate molecular targets emerging from these studies, together with their cellular context, age‐associated regulation, functional class and current level of experimental validation, are summarized in Table 2.

**TABLE 2:** Candidate molecular targets identified by single‐cell and spatial transcriptomic studies of skin aging.

Author	Target/pathway	Cell type/compartment	Functional class	Age‐associated change	Evidence level
Zou et al., 2021 [10]	KLF6	Basal keratinocytes	Transcription factor	Downregulated with aging	In vitro functional validation
	KLF4	Basal keratinocytes	Transcription factor	Downregulated with aging	Transcriptomic association only
	KLF5	Basal keratinocytes	Transcription factor	Downregulated with aging	Transcriptomic association only
	SOX9	Basal keratinocytes	Transcription factor	Downregulated with aging	Transcriptomic association only
	HES1	Dermal fibroblasts	Transcription factor	Downregulated with aging	In vitro functional validation
	IER2	Dermal fibroblasts	Transcription factor	Downregulated with aging	In vitro functional validation
	ID1	Dermal fibroblasts	Transcription factor	Downregulated with aging	Transcriptomic association only
	ID3	Dermal fibroblasts	Transcription factor	Downregulated with aging	In vitro functional validation
	TSC22D1	Dermal fibroblasts	Transcription factor	Downregulated with aging	In vitro functional validation
	GADD45B	Dermal fibroblasts	Transcription factor	Downregulated with aging	Transcriptomic association only
	ZFP36	Dermal fibroblasts	Transcription factor	Downregulated with aging	Transcriptomic association only
	JAG1/DLL1–NOTCH3	Dermal fibroblasts/endothelial cells ↔ basal keratinocytes	Intercellular signalling pathway	Downregulated with aging	Computational inference
Li et al., 2026 [61]	FASN	Sebaceous gland/epidermal compartment	Metabolic regulator	Downregulated with aging	In vitro +3D functional validation
	FDPS	Sebaceous gland/epidermal compartment	Metabolic regulator	Downregulated with aging	In vitro +3D functional validation
	FADS1	Sebaceous gland	Metabolic regulator	Downregulated with aging	Tissue‐level validation
	FADS2	Sebaceous gland/epidermal compartment	Metabolic regulator	Downregulated with aging	Tissue‐level validation; in vitro testing (negative)
	ELOVL3	Sebaceous gland	Metabolic regulator	Downregulated with aging	Tissue‐level validation
	APOE	Sebaceous gland	Metabolic regulator	Downregulated with aging	Tissue‐level validation
	c‐FOS	Epidermis	Transcription factor	Downregulated with aging	Tissue‐level validation
	JUNB	Epidermis	Transcription factor	Downregulated with aging	Tissue‐level validation
	MAFB	Epidermis	Transcription factor	Downregulated with aging	Transcriptomic association only
	S100A7	Multiple spatial clusters	Intercellular signalling/inflammatory factor	Upregulated with aging	Transcriptomic association only
	S100A8	Multiple spatial clusters	Intercellular signalling/inflammatory factor	Upregulated with aging	Transcriptomic association only
	S100A9	Multiple spatial clusters	Intercellular signalling/inflammatory factor	Upregulated with aging	Tissue‐level validation
Li et al., 2026 [63]	ATF3	Basal keratinocytes	Transcription factor	Downregulated with aging	In vitro + ex vivo + in vivo functional validation
	KLF6	Basal keratinocytes	Transcription factor	Downregulated with aging	In vitro functional validation
	TXNRD1	Basal keratinocytes	Metabolic regulator	Downregulated with aging	In vitro functional validation
	COL17A1/COL7A1–integrin complexes	Dermal fibroblasts ↔ basal keratinocytes	Intercellular signalling pathway	Downregulated with aging	Computational inference
	CXCL2	Dermal fibroblasts ↔ basal keratinocytes	Intercellular signalling pathway	Upregulated with aging	Computational inference + in vitro functional support
Pan et al., 2026 [65]	TNF pathway	Fibroblast–immune/macrophage–T‐cell interactions	Intercellular signalling pathway	Upregulated with aging	Computational inference
	CXCL pathway	Fibroblast–immune/macrophage–T‐cell interactions	Intercellular signalling pathway	Upregulated with aging	Computational inference
	MIF pathway	Fibroblast–immune/macrophage–T‐cell interactions	Intercellular signalling pathway	Upregulated with aging	Computational inference
Ahlers et al., 2021 [59]	HES1	Dermal sheath (DS) cells	Transcription factor	Downregulated with aging	In vitro functional validation
	INHBA	Dermal sheath (DS) cells	Intercellular signalling pathway	Downregulated with aging	In vitro +3D functional validation
	MDK	Dermal sheath (DS) cells	Intercellular signalling pathway	Downregulated with aging	In vitro functional validation
	RBP4	Dermal sheath (DS) cells	Intercellular signalling pathway	Downregulated with aging	In vitro functional validation
Nakamizo et al., 2025 [58]	SERPINB3	Upper epidermis	Intercellular signalling/inflammatory factor	Upregulated with aging	Tissue‐level validation
	CD55	Epidermis; specific cell population not individually reported	Intercellular signalling/inflammatory factor	Upregulated with aging	Transcriptomic association only
	RORA	Epidermis; specific cell population not individually reported	Intercellular signalling/inflammatory factor	Upregulated with aging	Transcriptomic association only
	MIF	Epidermis; specific cell population not individually reported	Intercellular signalling/inflammatory factor	Upregulated with aging	Transcriptomic association only
	TXNIP	Epidermis; specific cell population not individually reported	Metabolic regulator	Downregulated with aging	Transcriptomic association only

*Note:* Evidence level reflects the highest level of validation reported for each target/pathway in the cited studies; computational ligand–receptor analyses are classified as correlative inference rather than functional validation.

### 4.1 Metabolic Regulators of Skin Aging

Recent single‐cell and spatial transcriptomic studies have identified metabolic remodelling as a fundamental component of skin aging. High‐resolution spatial transcriptomics demonstrated marked downregulation of lipid synthesis and fatty acid elongation genes in the aged stratum granulosum, including *FASN*, *FDPS*, *FADS1*, *FADS2*, *ELOVL3* and *APOE*, coinciding with reduced epidermal differentiation gene expression [61]. Pharmacological inhibition of FASN and FDPS reduced keratinocyte proliferation and epidermal thickness, demonstrating that epidermal structure requires active lipid metabolic programmes.

Additional single‐cell studies indicate that aging keratinocytes downregulate redox regulators such as TXNRD1 and TXNIP [58, 63], while fibroblasts, endothelial cells and immune populations show widespread alterations in mitochondrial oxidative phosphorylation and energy metabolism [60, 64]. Independent datasets similarly report impaired oxidative stress defence together with endoplasmic reticulum stress and altered mTOR signalling [58, 60, 64], suggesting that restoring cellular metabolic fitness may be an important avenue for future rejuvenation strategies.

### 4.2 Transcription Factors Regulating Cell Identity and Regenerative Capacity

Among the molecular regulators emerging from aging atlases, transcription factors have attracted particular attention because of their ability to orchestrate broad gene expression programmes controlling cell identity, proliferation, differentiation and tissue regeneration.

One of the first observations from single‐cell analyses was the coordinated decline of transcriptional programmes associated with epidermal identity. Zou and colleagues reported that epithelial transcription factors, including Krüppel‐like factor family members (KLF4, KLF5, KLF6) and SOX9, decreased within basal keratinocyte populations during aging [10, 66, 67, 68]. Functional experiments showed that KLF6 silencing in primary human keratinocytes impaired self‐renewal and induced a senescence‐associated, inflammatory phenotype, supporting a direct role for KLF6 in epidermal homeostasis.

Transcriptional remodelling was similarly evident within the dermal compartment. *HES1* was identified as one of the genes most consistently downregulated during aging in dermal fibroblasts [10]. HES1 is a canonical downstream effector of Notch signalling that regulates stem cell maintenance, cell fate decisions and cell differentiation in the skin [69, 70, 71]. Its depletion promotes senescence, while restoring HES1 signalling partially rescues age‐associated phenotypes. Additional factors, including IER2, ID1, ID3, TSC22D1, GADD45B and ZFP36, were coordinately reduced, indicating widespread deterioration of fibroblast identity [10].

More recently, a preprint study identified the transcription factor ATF3 as a candidate regulator of epidermal stem cell function during aging [63]. ATF3 is an adaptive stress‐responsive transcription factor rapidly induced following tissue injury and environmental stress [72, 73]. ATF3 expression progressively declined in aged basal keratinocytes and was associated with impaired regenerative capacity, and restoring ATF3 expression improved multiple features of aged human skin, including epidermal stem cell proliferation, reduced senescence and increased fibroblast collagen production.

Spatial transcriptomic analyses also identified reduced activity of AP‐1 family transcription factor regulons, including JUNB, FOS and MAFB, in aged human epidermis [61]. Although their functional contribution was not directly investigated, AP‐1 factors are well‐established mediators of keratinocyte proliferation, differentiation, stress responses and epidermal repair, suggesting that their coordinated downregulation may contribute to the decline of epidermal homeostasis during aging [74, 75, 76].

### 4.3 Intercellular Signalling Pathways Coordinating Tissue Homeostasis

Cutaneous homeostasis relies on continuous bidirectional signalling between epidermal, dermal, immune, vascular and appendage‐associated cell populations [77, 78, 79]. Single‐cell and spatial transcriptomic approaches have predicted that aging weakens these signalling circuits, suggesting that restoration of tissue homeostasis may require not only rejuvenation of individual cell populations but also re‐establishment of the molecular interaction between distinct skin compartments.

Ligand‐receptor analyses of single‐cell datasets by Zou and colleagues identified a reduction of JAG1/DLL1‐NOTCH3 interactions between fibroblasts, endothelial cells and basal keratinocytes during aging, with downregulation of HES1 in dermal fibroblasts, suggesting deterioration of the NOTCH‐HES1 axis contributes to loss of dermal homeostasis [10]. High‐resolution spatial transcriptomics further inferred a global reduction in dermal‐epidermal communication, identifying WNT and NOTCH signalling as major pathways attenuated in aged human skin [61]. However, the Notch signalling pathway represents a potentially double‐edged therapeutic target, as Notch signalling plays context‐dependent roles in epidermal homeostasis, differentiation and tumour suppression, requiring careful consideration when therapeutically modulating this pathway [80, 81, 82].

Beyond pathway‐level analyses, aging atlases have identified specific secreted molecules capable of functionally restoring tissue properties. Ahlers et al. found that dermal sheath cells produce a paracrine secretome enriched in Midkine (MDK), Retinol Binding Protein 4 (RBP4) and Activin A (INHBA) [59]. Recombinant Activin A stimulated keratinocyte proliferation, enhanced procollagen production and restored epidermal thickness and collagen synthesis in aged skin equivalents; MDK and RBP4 did not reproduce Activin A's broader regenerative effects.

Additional aging atlases have expanded the repertoire of candidate signalling molecules involved in tissue communication. Ligand‐receptor analyses predicted age‐sensitive interactions involving COL17A1/COL7A1‐integrin complexes and the ATF3‐regulated chemokine CXCL2 [63]. In the same preprint study, functional experiments further showed that conditioned medium from young keratinocytes or aged keratinocytes following ATF3 mRNA delivery, reduced fibroblast senescence while enhancing collagen and elastin production. Reduced predicted signalling via BMPs, FGFs, kallikreins, protein S, melanocortins and pleiotrophin between keratinocyte and epidermal populations was also identified in aged epidermis [58].

Beyond the decline of developmental signalling, multiple aging atlases revealed increased inflammatory mediators in aged skin, including S100 family members and genes such as *CD55*, *MIF*, *SERPINB3*, *RORA* and *SFN*, while regulators *PTPRCAP* and *CD48* were reduced in fibroblasts [10, 58, 64]. Spatial transcriptomic analyses implicated TNF signalling as central to CCL19^+^ perivascular fibroblasts [9], while integrative single‐cell analysis identified an aging‐associated SFRP2^+^ fibroblast population with predicted increase in TNF, CXCL and MIF signalling [65], reflecting fibroblast‐immune remodelling within a chronic inflammatory microenvironment.

Collectively, these findings consistently predict that aging is accompanied by deterioration of the molecular networks that coordinate communication among skin cell populations. The successful functional validation of Activin A and CXCL2‐associated epidermal‐dermal signalling indicates that restoration of tissue communication may represent a complementary therapeutic strategy to direct cellular rejuvenation.

## 5 Current Rejuvenation Strategies: Strengths and Limitations

The identification of transcriptional, metabolic and intercellular signalling networks underlying skin aging provides a useful framework for re‐evaluating current rejuvenation strategies [83], ranging from topical compounds and minimally invasive aesthetic procedures to regenerative approaches targeting senescence or epigenetic reprogramming [84, 85, 86, 87]. Despite remarkable advances in aesthetic and regenerative dermatology, current interventions differ substantially in their mechanisms of action, ranging from modulation of defined molecular pathways to structural replacement and induction of endogenous tissue remodelling.

### 5.1 Topical Therapies

Topical agents remain the mainstay of skin rejuvenation due to their accessibility, favourable safety profile and extensive clinical experience [85, 88, 89, 90]. Retinoids are considered the gold standard for topical anti‐aging therapy [26, 91, 92]. By activating retinoic acid receptors, they promote epidermal turnover, stimulate collagen synthesis, inhibit matrix metalloproteinase activity and improve dermal extracellular matrix organization, reducing fine wrinkles and improving pigmentation and texture [26, 85, 90, 91, 92, 93].

Numerous additional compounds have been incorporated into topical formulations, including antioxidants such as vitamin C, vitamin E and coenzyme Q10, along with niacinamide, growth‐promoting peptides and hyaluronic acid [94, 95, 96, 97, 98, 99], aiming to reduce oxidative stress, improve hydration, stimulate matrix production or enhance barrier function. Their efficacy is often limited, however, by poor skin penetration, chemical instability, variable bioavailability and modest biological activity.

### 5.2 Energy‐Based and Regenerative Procedures

Over the past two decades, the therapeutic options available for skin rejuvenation have substantially expanded. Fractional lasers, intense pulsed light, radiofrequency, ultrasound‐based devices and microneedling induce controlled tissue injury that activates endogenous wound healing programmes, stimulating fibroblast activity, collagen remodelling and new collagen deposition [100, 101, 102, 103].

Injectable therapies, including hyaluronic acid fillers, platelet‐rich plasma (PRP) and bio‐stimulatory agents, similarly seek to restore tissue volume and improve dermal architecture by enhancing extracellular matrix remodelling or providing trophic factors that support tissue repair [104, 105, 106].

These approaches largely rely on inducing tissue remodelling or replacing structural components rather than correcting the molecular alterations that accumulate during aging. As a result, repeated treatments are generally required to maintain benefit and more intensive interventions may carry procedure‐related risks, including pigmentary alterations, scarring or infection [107].

### 5.3 Senolytic and Senomorphic Approaches

Cellular senescence has emerged as an important therapeutic target in skin aging. Senescent cells accumulate with age and drive tissue dysfunction by secreting pro‐inflammatory cytokines, chemokines, growth factors and matrix‐remodelling enzymes, collectively termed the SASP [108, 109]. Senotherapeutic strategies broadly include elimination of senescent cells through senolytics [110, 111, 112] and senomorphics, which suppress detrimental features of the senescent phenotype, particularly the SASP, without inducing cell death [113]. Pharmacological clearance of senescent cells improves tissue function and delays age‐associated phenotypes in preclinical models [8, 114]. Despite encouraging preclinical evidence, the clinical translation of topical senolytics for skin aging remains limited [115, 116, 117]. Senomorphic approaches have progressed somewhat further, with topical rapamycin showing reductions in senescence markers together with improvements in histological and clinical features of aged human skin in an exploratory clinical study [118]. Nevertheless, larger controlled studies are required to establish the long‐term efficacy and safety of topical senotherapeutic strategies.

### 5.4 Partial Cellular Reprogramming

Advances in cellular reprogramming have introduced the possibility of reversing age‐associated molecular alterations at the transcriptional level [119, 120]. Transient expression of the Yamanaka factors (OCT4, SOX2, KLF4 and c‐MYC) has been shown to partially reset epigenetic age while preserving cellular identity, raising considerable interest in partial reprogramming as a rejuvenation strategy [120, 121].

Although these targeted approaches have demonstrated remarkable regenerative potential in experimental systems, important concerns remain regarding genomic instability and delivery strategies, limiting their current clinical applicability [86, 87, 122, 123].

## 6 Emerging Nucleic Acid Therapeutics for Skin Rejuvenation

The expanding molecular resolution of skin aging has created opportunities for therapeutic modalities capable of complementing established rejuvenation approaches. Many candidate targets identified by single‐cell and spatial transcriptomic studies are transcription factors, metabolic regulators or secreted signalling molecules not easily manipulated using conventional pharmacological agents. Nucleic acid therapeutics have emerged as tools for directly modulating gene expression rather than alleviating downstream consequences [124, 125]. RNAi, antisense oligonucleotides (ASOs) and mRNA‐based therapies enable precise gene modulation but differ in mechanism and application (Figure 3).

**FIGURE 3:**
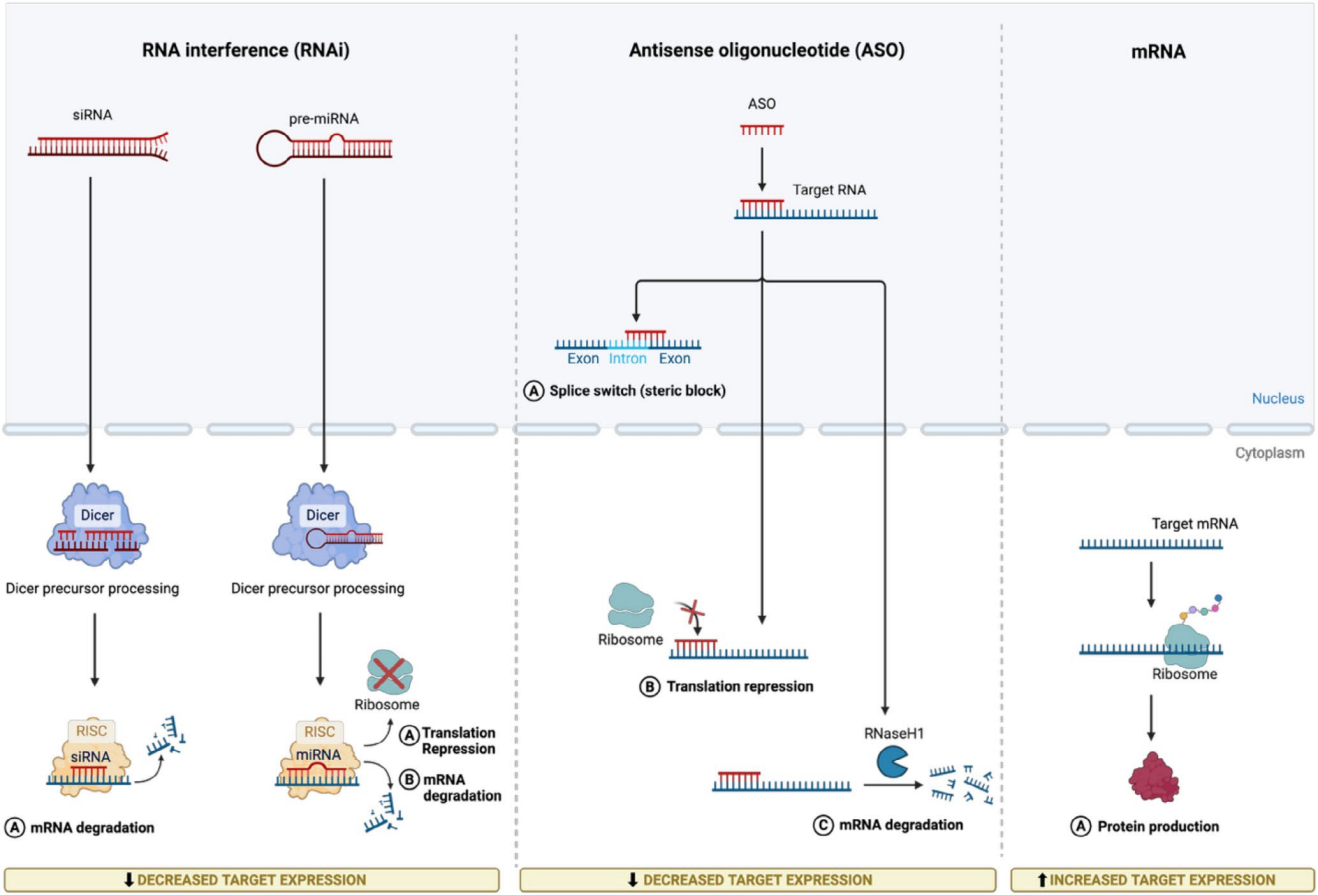
Mechanisms of action of nucleic acid therapeutics. Schematic representation of the three principal classes of nucleic acid therapeutics. RNA interference (RNAi) employs small interfering RNAs (siRNAs) or microRNA (miRNA)‐based approaches that associate with the RNA‐induced silencing complex (RISC) to suppress target gene expression through mRNA cleavage or translational repression. Antisense oligonucleotides (ASOs) hybridize with complementary RNA sequences to modulate gene expression by altering pre‐mRNA splicing, blocking translation or inducing RNase H‐mediated degradation. Messenger RNA (mRNA) therapeutics transiently restore protein expression by delivering synthetic mRNA to the cytoplasm, where it is translated into protein without genomic integration. Image created using BioRender (BioRender.com).

### 6.1 RNA Interference

RNA interference (RNAi) exploits endogenous post‐transcriptional silencing to selectively reduce gene expression through short interfering RNAs (siRNAs) or microRNA‐based approaches [126, 127]. siRNAs guide the RNA‐induced silencing complex (RISC) to a complementary target mRNA, causing its cleavage and degradation [128, 129]. miRNAs likewise recognize targets through sequence complementarity and rely on RISC [126, 130], but unlike siRNAs, which primarily cause direct cleavage of mRNA [124, 131], miRNAs mainly inhibit translation and destabilize target transcripts [126, 132].

RNAi‐based therapeutics have also attracted interest for cutaneous disorders, including CTGF‐targeting siRNAs for hypertrophic scars and keloids, intradermal STAT3 siRNA for melanoma and topical co‐delivery of anti‐STAT3 and anti‐TNFα siRNAs for psoriasis [133, 134, 135]. siRNA‐mediated knockdown of SFRP4 also attenuated skin aging by suppressing the SASP and preserving dermal structure in preclinical models [136] and advances in delivery platforms, including lipophilic siRNAs and STAR particle technology, are expanding the feasibility of local cutaneous RNAi [137, 138, 139].

Beyond skin aging, therapeutic modulation of miRNAs in the skin has been more extensively explored in regenerative and pathological contexts, providing evidence for the feasibility of local miRNA‐based interventions. For example, delivery of miR‐21 mimics has been used to accelerate wound healing, while delivery of miR‐29b mimics attenuated scar formation via TGF‐β modulation [140, 141]. More recently, a miRNA cocktail comprising miR‐19b‐3p, miR‐132‐3p and miR‐31‐5p with anti‐miR‐92a significantly enhanced repair of diabetic wounds by inhibiting local inflammation while promoting epidermal regeneration, neovascularization and granulation tissue formation [142]. Although these studies do not directly address skin aging, they demonstrate that miRNA‐based interventions can modulate several biological processes that are also compromised in aged skin, including regenerative capacity, inflammatory signalling and tissue remodelling.

While the ability of siRNAs and miRNAs to selectively silence individual genes and modulate broader regulatory networks offers considerable therapeutic potential, further work is required to define the optimal molecular targets and delivery strategies capable of achieving durable rejuvenation of aged skin.

### 6.2 Antisense Oligonucleotides

Antisense oligonucleotides (ASOs) constitute another major class of nucleic acid therapeutics designed to modulate gene expression through sequence‐specific hybridization with target RNA molecules [125, 143, 144]. Unlike RNAi, which relies on endogenous RNA silencing machinery, ASOs directly bind complementary RNA sequences and exert their biological effects through RNase H‐mediated degradation, inhibition of mRNA translation, modulation of pre‐mRNA splicing and interference with RNA processing or stability [145].

ASO‐based therapeutics have also been investigated for cutaneous aging and other disorders. Topical delivery of a peptide nucleic acid (PNA)‐based antisense oligonucleotide targeting MMP1 improved wrinkle appearance, elasticity, hydration and dermal density [146]. QR‐313, a splice‐switching ASO inducing exon 73 skipping in COL7A1, restored collagen VII expression and improved wound healing in dystrophic epidermolysis bullosa models [147]. Topical delivery of an ASO targeting IL‐10 also ameliorated dermatitis‐like lesions in NC/Nga mice [148].

Although the dermatological application of ASOs remains relatively limited, these studies demonstrate the versatility of this approach and its therapeutic potential for the treatment of several skin disorders.

### 6.3 Messenger RNA Therapeutics

Messenger RNA therapeutics fundamentally differ from RNAi and ASOs by enabling transient synthesis of functional proteins rather than suppressing endogenous gene expression. Several recent studies have instead exploited mRNA therapeutics to restore structural and regulatory proteins implicated in skin aging.

A major application of mRNA therapeutics has focused on restoring extracellular matrix components lost during aging. COL1A1 mRNA delivered within engineered extracellular vesicles promoted collagen production within the dermal compartment and reduced wrinkle formation in photoaged skin [149], while intradermal delivery of COL3A1 mRNA restored dermal architecture, barrier function, dermal thickness and collagen content while reducing oxidative stress and senescence in photoaging models [14]. Tropoelastin (TE) mRNA likewise induced de novo elastogenesis within the dermis and improved photoaged and naturally aged skin [150].

Beyond structural proteins, mRNA therapeutics have also been used to restore regulatory molecules whose expression declines with age. In a preprint study, ATF3 mRNA delivery rescued age‐associated loss of ATF3 in basal epidermal stem cells, in which its decline was originally identified, reduced senescence, enhanced stem‐cell proliferation and promoted fibroblast collagen production through improved epidermal–dermal communication [63]. Similarly, EV‐mediated delivery of Gstm2 mRNA within the dermis restored antioxidant defences in aged dermal fibroblasts, re‐established dermal‐epidermal communication and accelerated wound healing in aged mice [151].

Collectively, these studies illustrate the capability of mRNA therapeutics to transiently replace proteins that are lost or diminished during aging, thereby expanding the spectrum of therapeutically accessible targets beyond those achievable through gene‐silencing approaches.

### 6.4 Comparative Advantages of mRNA Therapeutics

mRNA therapeutics enable direct production of a desired protein, thereby providing a complementary strategy for restoring factors that are reduced or lost during aging. This is attractive for skin aging since transcriptomic analyses suggest most candidate targets are lost rather than aberrantly activated.

The suitability of mRNA as a therapeutic modality ultimately depends on the nature of the molecular target and should be also considered alongside alternative strategies. Small molecules offer well‐established and readily scalable pharmacological strategies when suitable druggable proteins or pathways are available, but many age‐associated factors identified by single‐cell atlases, particularly transcriptional regulators, are not readily responsive to conventional small‐molecule modulation. Recombinant proteins may represent a more direct strategy for extracellular factors, as demonstrated by the regenerative effects of recombinant Activin A [59]. However, protein‐based approaches are less suited to restoring intracellular regulators, including transcription factors, because of the limited ability of proteins to cross cellular membranes and reach intracellular targets [152, 153].

Gene therapy provides an alternative strategy for restoring protein expression. In particular, adeno‐associated viral (AAV) vectors can support substantially longer‐lasting expression than mRNA, which may be advantageous when sustained correction is required [154, 155]. However, prolonged expression may be less desirable for the modulation of regulatory proteins whose biological effects are strongly dependent on dose, duration and cellular context. In addition, pre‐existing or treatment‐induced immunity against AAV capsids can limit vector administration and complicate redosing [156, 157].

In this setting, mRNA occupies a complementary therapeutic niche by enabling transient and dose‐dependent protein expression without introducing a persistent genetic expression cassette. This combination may be particularly valuable for intracellular proteins that cannot be directly replaced pharmacologically and for regulatory factors for which reversible modulation is preferable to sustained expression. Thus, rather than representing a universally superior modality, mRNA provides a flexible approach for extending therapeutic intervention to selected atlas‐derived targets that are difficult to address using recombinant proteins, small molecules or persistent gene‐transfer strategies.

### 6.5 Engineering Therapeutic mRNA for Cutaneous Applications

The therapeutic performance of synthetic mRNA depends not only on the encoded protein but also on the molecular design and purity of the RNA itself. Several structural elements can be engineered to optimize translation and stability, including the 5′ cap, 5′ and 3′ untranslated regions (UTRs), coding sequence and poly(A) tail [158]. The 5′ cap is essential for efficient translation initiation and protects the transcript from exonucleolytic degradation, whereas UTR sequence and architecture influence transcript stability, ribosome recruitment and translational efficiency [158, 159, 160]. Similarly, optimization of the coding sequence and poly(A) tail can further modulate mRNA stability [158, 161].

A second critical consideration is innate immune recognition of exogenous RNA. Incorporation of modified nucleosides, particularly N1‐methylpseudouridine, can reduce recognition by innate RNA‐sensing pathways while supporting efficient protein production [162]. In addition, in vitro transcription can generate double‐stranded RNA (dsRNA) by‐products that activate innate immune sensors and interfere with translation [163, 164]. Removal of these contaminants through appropriate purification strategies can therefore substantially improve both mRNA expression and tolerability [163, 165]. Together, nucleoside modification, sequence and structural optimization and stringent control of RNA purity determine the magnitude and kinetics of protein expression while limiting unwanted innate immune activation. These parameters are particularly relevant for cutaneous applications, where local immune surveillance and the potential requirement for repeated administration make control of reactogenicity and expression kinetics central considerations for therapeutic design.

## 7 Cutaneous Delivery Strategies for mRNA Therapeutics

The therapeutic potential of mRNA is intrinsically linked to the development of efficient delivery systems capable of overcoming cutaneous barriers. Naked mRNA is highly susceptible to enzymatic degradation and poor cellular uptake [166] and the stratum corneum severely limits penetration into deeper skin layers [167, 168]. Cutaneous mRNA therapy therefore relies on delivery platforms that protect mRNA from degradation, facilitate uptake and achieve efficient protein expression within target cells. Carrier systems, including lipid nanoparticles (LNPs) and extracellular vesicles (EVs), protect mRNA from degradation and facilitate cellular uptake and intracellular release, whereas physical delivery methods such as microneedles (MNs) primarily overcome the stratum corneum barrier and enable local deposition within viable skin layers. Importantly, these approaches are not mutually exclusive, as microneedles can be combined with mRNA carrier systems to facilitate their cutaneous administration (Figure 4).

**FIGURE 4:**
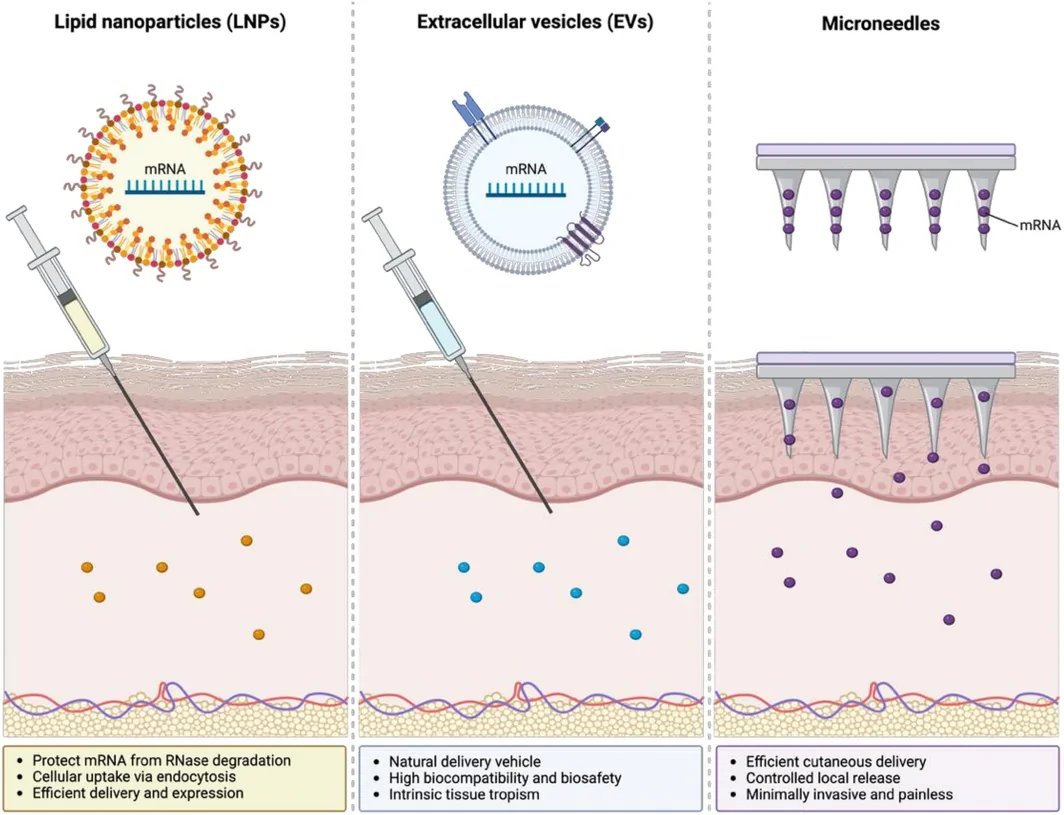
Major carrier systems and physical strategies for cutaneous mRNA delivery. Schematic overview of the approaches currently investigated for local cutaneous mRNA delivery. Lipid nanoparticles (LNPs) protect mRNA from enzymatic degradation, facilitate cellular uptake and promote intracellular delivery. Extracellular vesicles (EVs) exploit endogenous lipid vesicles as natural carriers characterized by high biocompatibility and intrinsic tissue tropism. Microneedles (MNs) bypass the stratum corneum through minimally invasive microchannels, minimizing tissue damage and discomfort. Image created using BioRender (BioRender.com).

### 7.1 Lipid Nanoparticles

Lipid nanoparticles (LNPs) represent the gold standard technology for clinical nucleic acid therapeutics and they are typically composed of four major components: ionizable lipids, helper phospholipids, cholesterol and polyethylene glycol (PEG)‐lipids [15, 169, 170]. Ionizable lipids enable electrostatic complexation with negatively charged mRNA [171, 172], helper phospholipids and cholesterol stabilize the nanoparticle and promote membrane fusion [173], while PEG‐lipids improve colloidal stability and circulation time [174]. Following endocytic uptake, endosomal acidification protonates the ionizable lipids, releasing mRNA into the cytoplasm for translation [169].

This technology has been employed also in skin aging, where LNP‐mediated delivery has been successfully used to restore the expression of structural extracellular matrix proteins, including COL3A1 and tropoelastin, demonstrating the feasibility of local mRNA replacement strategies in aged skin [14, 150].

Despite these advantages, challenges remain: following local administration, achieving an optimal balance between nanoparticle diffusion and tissue retention is critical to maximize protein expression while minimizing local inflammatory responses [15], and future LNP formulations will likely require improved cell type‐specific targeting to maximize therapeutic efficacy while minimizing off‐target effects [175].

### 7.2 Extracellular Vesicles

Extracellular vesicles (EVs) have recently emerged as a promising alternative to synthetic nanocarriers for mRNA delivery. EVs are naturally secreted lipid bilayer‐enclosed vesicles that mediate intercellular communication by transporting proteins, lipids and nucleic acids between cells [176, 177]. Their endogenous origin confers advantages over synthetic systems, including high biocompatibility, low immunogenicity and intrinsic stability [178], with natural tissue tropism allowing interaction with target cells via membrane fusion or endocytic uptake [179].

The therapeutic potential of EV‐mediated mRNA delivery has recently been explored in skin rejuvenation. Engineered EVs have been used to deliver COL1A1 and Gstm2 mRNAs, demonstrating efficient local protein replacement and tissue regeneration in experimental models of skin aging [149, 151]. These findings suggest that EVs combine efficient mRNA delivery with the biological advantages of endogenous vesicles, though challenges related to scalable production, cargo loading and manufacturing standardization remain [180].

### 7.3 Microneedle‐Based Delivery

Among physical delivery strategies, microneedles are one of the most promising approaches for local mRNA administration to the skin. By creating microscopic, transient channels across the stratum corneum, microneedles enable direct deposition of mRNA into viable epidermal and dermal layers while minimizing pain and tissue damage [181, 182, 183]. Localized delivery also minimizes systemic exposure and permits repeated administration, making MNs attractive for chronic dermatological conditions [183]. MNs can therefore serve as an administration interface for carrier‐based formulations, including LNP‐ or EV‐encapsulated mRNA.

The feasibility of MN‐mediated mRNA delivery was explored in a recent preprint study showing rapid local ATF3 protein expression following intradermal administration of synthetic mRNA, leading to skin regeneration [63]. These studies highlight microneedles as a promising strategy for local mRNA administration, though further optimization of microneedle fabrication, mRNA loading and stability and large‐scale manufacturing will be required for clinical translation [183, 184].

## 8 Major Open Questions

The rapid evolution of single‐cell and spatial transcriptomic technologies, together with advances in RNA‐based therapeutics, has opened unprecedented opportunities for mechanism‐driven skin rejuvenation. Nevertheless, several fundamental questions remain unresolved and will ultimately determine the clinical translation of these emerging strategies.

A first challenge concerns target selection, distinguishing causal drivers of skin aging from secondary alterations. Aging atlases have identified numerous candidate regulators, but it remains unclear which changes actively drive tissue deterioration versus merely reflecting downstream responses. Transcriptomic atlases also cannot capture regulatory events beyond gene transcription, since key processes can begin through post‐transcriptional and translational mechanisms before transcriptional changes occur [185]. Integrating transcriptomics with spatial proteomics, translatomics and functional perturbation studies will be essential to identify relevant targets.

An opportunity offered by mRNA therapeutics is their potential for combinatorial intervention. Skin aging involves coordinated alterations across multiple molecular pathways and cell populations, making it unlikely that modulation of a single target will fully restore tissue homeostasis. In this context, multiple mRNAs encoding complementary therapeutic factors can be co‐formulated within the same delivery platform, enabling simultaneous and transient modulation of distinct age‐associated pathways. This represents an important advantage of mRNA‐based approaches, although achieving controlled stoichiometry, efficient co‐delivery and appropriate cell‐type specificity will be critical for the development of such combinatorial strategies.

A critical consideration in translating aging atlases into mRNA‐based interventions is whether therapeutic delivery can reproduce the cell‐type specificity of the molecular alterations identified by single‐cell analyses. Most current cutaneous delivery approaches achieve localization at the skin‐compartment level rather than selective transfection of individual cell populations. This distinction is particularly relevant for intracellular regulators, such as transcription factors, whose biological activity is inherently dependent on cellular context and whose ectopic expression in non‐target populations may produce unintended effects. Thus, matching the cellular resolution of target discovery with comparable specificity in mRNA delivery represents an important challenge for the atlas‐to‐therapy framework.

Another unresolved question concerns the duration of therapeutic benefit. Although the transient nature of mRNA therapeutics may reduce risks associated with prolonged or irreversible expression [158, 186], it remains unknown whether short‐term restoration of key regulatory proteins is sufficient to induce durable tissue remodelling or whether repeated administration will be required. Moreover, for transcription factor‐based mRNA interventions, the transient nature of expression may help define a controllable therapeutic window but does not eliminate the risks associated with inappropriate or excessive activity. Establishing such a window will require careful control of dose and expression kinetics, cell‐type‐specific delivery and assessment of proliferative, differentiation and oncogenic consequences before these factors can be considered suitable rejuvenation targets. Defining the optimal therapeutic window, dosing schedule and treatment frequency will be an important step toward clinical implementation.

Available preclinical studies provide initial insight into the duration of response and feasibility of repeated administration. For example, in a photoaged mouse model, single administration of COL1A1 mRNA significantly improved wrinkle length and depth for up to 2 months, while monthly administration maintained improvements in wrinkle number and area throughout a 91‐day observation period [149]. Similarly, in a study of COL3A1 mRNA‐LNPs, a single intradermal administration increased dermal collagen III within 24 h, with elevated levels persisting for up to 28 days [14]. In the same study, five intradermal doses administered on days 1, 4, 7, 14, and 21 were well tolerated over the short observation period, with no detectable inflammatory infiltration in the skin or systemic toxicity [14]. These findings indicate that transient mRNA delivery can produce biological effects lasting substantially longer than the initial expression period and provide preliminary evidence for the feasibility of repeated administration. However, the available studies remain short‐term and do not establish the durability, optimal maintenance schedule or safety of chronic repeated dosing.

This remains an important consideration because repeated exposure may introduce immunological limitations that are less relevant to single‐dose treatment. The skin is an immunologically active organ and both the RNA cargo and delivery vehicle can contribute to innate immune activation and local reactogenicity. Although nucleoside modification, RNA purification and optimization of carrier composition can reduce these responses, repeated exposure to LNP components may also generate adaptive responses, including antibodies against PEG‐containing lipids, potentially altering tolerability and delivery efficiency upon subsequent administrations [163, 187]. Such effects have not yet been systematically evaluated in the context of long‐term mRNA‐based skin rejuvenation. A first‐in‐human Phase I study of SPOT‐mRNA01 (NCT06567119), an extracellular vesicle‐based formulation containing COL1A1 mRNA, has been registered specifically for skin aging [188]. The study is designed to evaluate safety and tolerability in healthy adults, together with local pharmacodynamic and skin‐related outcomes. Thus, clinical evidence regarding the safety, durability and efficacy of mRNA‐based skin rejuvenation remains to be established.

An additional challenge is patient heterogeneity. Skin aging is influenced not only by chronological aging but also by cumulative ultraviolet exposure, genetic background, gender and environmental and lifestyle factors [2, 189], so the molecular mechanisms underlying skin aging are unlikely to be identical across individuals. Future rejuvenation strategies will likely require molecular stratification to identify the most appropriate therapeutic targets for individual patients.

Collectively, addressing these questions will require integration of molecular biology, bioengineering, dermatology and clinical investigation. Their resolution will determine whether precision skin rejuvenation can successfully transition to routine clinical practice.

## 9 Conclusions and Perspectives

Advances in single‐cell and spatial transcriptomic technologies have transformed this field from a descriptive characterization of aging toward a mechanistic understanding of the cell type‐specific molecular alterations that impair tissue homeostasis. These atlases have revealed the cellular heterogeneity of aged skin and identified an expanding repertoire of candidate therapeutic targets previously inaccessible using conventional experimental approaches. These observations provide a high‐resolution molecular framework for understanding skin aging, although the causal and therapeutic relevance of many atlas‐derived alterations remains to be established.

In parallel, rapid progress in RNA engineering and delivery technologies has established nucleic acid therapeutics as a versatile platform for precise molecular intervention. mRNA therapeutics are particularly well suited to the molecular landscape emerging from skin aging atlases, enabling transient restoration of proteins lost during aging. Continuous improvements in delivery systems, including lipid nanoparticles, extracellular vesicles and microneedle‐based platforms, are further accelerating the clinical feasibility of local mRNA therapy. Preclinical studies have demonstrated that mRNA delivery can restore structural proteins, including collagens and elastin, as well as regulatory factors such as ATF3 and Gstm2, resulting in improvements in selected molecular, structural and functional features of aged or photoaged skin [14, 63, 149, 150, 151]. These studies provide proof‐of‐principle that transient protein restoration can produce biologically meaningful effects in the skin and that local delivery can be achieved using carrier systems and physical delivery strategies. Beyond these experimentally validated applications, aging atlases suggest additional therapeutic strategies that remain to be tested. For example, age‐associated loss of transcriptional regulators such as KLF6 and HES1 raises the possibility of restoring cell identity and regenerative programmes through transient mRNA delivery, while alterations in metabolic regulators suggest opportunities to modulate epidermal metabolism [10, 61].

Despite these advances, translation of molecular rejuvenation strategies will require overcoming not only biological and technological challenges, including target validation, patient stratification and long‐term clinical evaluation, but also important regulatory barriers. In particular, injectable approaches that reach the vascularized dermal compartment may fall within medicinal‐product rather than cosmetic regulatory frameworks, substantially increasing the requirements for clinical development, manufacturing, safety evaluation and associated costs. An additional challenge is that skin aging itself is generally not recognized as a disease, complicating the design of clinical trials and regulatory pathways for interventions aimed primarily at rejuvenation. A potentially more tractable translational strategy may therefore be to target specific age‐associated cutaneous diseases, providing defined clinical endpoints while simultaneously testing the therapeutic value of targeting age‐related molecular alterations. Manufacturing and chemistry, manufacturing and controls (CMC) represent additional challenges for clinical translation. mRNA‐based products require stringent control of RNA identity, integrity, purity, potency and residual process‐related impurities, while carrier‐based formulations additionally require reproducible encapsulation efficiency, particle composition and size and functional delivery properties. Formulation stability, storage conditions and batch‐to‐batch consistency will also need to be established, particularly for products intended for repeated administration.

Addressing these biological, technological and regulatory challenges will be essential for translating the convergence of high‐resolution aging atlases and RNA therapeutics into clinically viable interventions. Future precision approaches guided by cellular atlases are expected to become an increasingly important component of regenerative dermatology.

## Author Contributions

F.B., S.S. and A.M. designed the manuscript. F.B. wrote the manuscript and prepared the figures. S.S., K.T. and A.M. reviewed the manuscript for important intellectual content. All authors have read and agreed to the published version of the manuscript.

## Funding

A.M. is funded by the U.S. Department of Health and Human Services, NIH, National Institute on Aging (U.S. National Institute on Aging)—1P01AG071463‐02.

## Ethics Statement

The authors have nothing to report.

## Consent

The authors have nothing to report.

## Conflicts of Interest

A.M. is a co‐founder (with equity) of New Frontier Bio, a consumer health company developing skincare and anti‐aging products and has equity in DermBiont, a private company advancing targeted topical therapeutics for dermatological indications. K.T. has a financial interest in New Frontier Bio.

## Data Availability

Data sharing not applicable to this article as no datasets were generated or analysed during the current study.
